# Grayson’s Laryngology

**Published:** 1902-10

**Authors:** 


Grayson's Laryngology.—A treatise on the diseases of the throat, nose and the associated affections of the ear. By Charles P. Grayson, M. D., Lecturer on and Instructor in Laryngology in the Medical Department, University of Pennsylvania. In one octavo volume of 540 pages, with 129 engravings and 8 colored plates. Cloth, $3.50, net. Lea Brothers & Co., Philadelphia and New York, 1902.

The author begins with a chapter on the selection and care of instruments in which he includes a description and illustration of every device and appliance which he deems necessary for the purpose of examination and treatment in the practice of rhinolaryngology. His second chapter deals with the examination of the patient. In this he clearly and tersely outlines the various steps that should be followed in the making of an examination. Then, in order, are taken up the anatomy and physiology of the nose, throat and ear, and, the subjects of inflammations, neuroses, neoplasms, affections of the septum, etc.

Perhaps the most valuable feature of the work are to be found in its sections on treatment. By recommending but one plan of procedure in each instance, and that the one which the author has, during his many years of experience as teacher and practitioner, found to be the best, he never leaves the reader in doubt as to what is best to be done in a given case, and as to the method by which it may most easily be accomplished.

The book is well illustrated and excellently printed and bound. It deserves a large sale, which it will no doubt have. No work on the subject deserves greater popularity with the profession.

